# Assessing Nurses’ Knowledge and Attitudes towards Cancer Pain Management in Oman

**DOI:** 10.3390/cancers15153925

**Published:** 2023-08-02

**Authors:** Adhari Al Zaabi, Maeen Al-Saadi, Husain Alaswami, Atika Al-Musalami

**Affiliations:** 1 Department of Human and Clinical Anatomy, College of Medicine, Sultan Qaboos University, Muscat 123, Oman; 2 College of Medicine, Sultan Qaboos University, Muscat 123, Oman; s130684@student.squ.edu.om (M.A.-S.); s132017@student.squ.edu.om (H.A.); 3 Oncology Center, Royal Hospital, Ministry of Health, Muscat 393, Oman

**Keywords:** cancer pain, advanced stage, nurses, palliative, knowledge, attitude

## Abstract

**Simple Summary:**

Nurses’ knowledge, expertise, and attitudes significantly impact pain management quality. This study showed that 50% had poor knowledge and attitudes, with a significant association between knowledge and education level, experience caring for cancer patients, and prior pain management training. Low knowledge and attitude pose significant challenges. Adequate training in cancer pain management is crucial to improve pain control and enhance the quality of life for cancer patients.

**Abstract:**

Cancer care in the Gulf Cooperation Countries, including Oman, faces challenges due to increasing incidence and late-stage diagnoses. Cancer patients at later stages suffer complex symptoms, pain being a prominent one. Access to adequate pain relief is a global problem, including in the Middle East, where palliative care is lacking. Nurses play a crucial role in pain assessment and management but often lack the necessary training, resulting in inadequate relief and prolonged hospital stays. This study aims to examine the knowledge and attitudes of nurses in a national cancer center toward the management of cancer pain, with the goal of identifying any gaps in their knowledge. This is a cross-sectional descriptive study conducted among nurses at the national cancer center in the Royal Hospital. The Nurses’ Knowledge and Attitudes Survey Regarding Pain (NKASRP) was used to determine the pain-related knowledge and attitudes of the nurses. Out of 150 registered nurses, 118 participated in this study (78% response rate). The mean NKASRP score was 49.6%. Half of the participants (50%) had a poor level of knowledge and attitude, 46% had fair knowledge and attitude, and only five participants (4%) had a good level of knowledge and attitude. A statistically significant association existed between knowledge and education level, years of experience caring for cancer patients, and prior pain management training (*p* < 0.05). A low level of knowledge and attitude among nurses in cancer pain management is a significant challenge in providing comprehensive cancer care. Adequate training of nurses in cancer pain management is essential to providing effective pain management and improving the quality of life of cancer patients.

## 1. Introduction

The Gulf Cooperation Countries (GCCs), including Oman, are committed to improving the well-being of cancer patients through the implementation of advanced treatments and updated management guidelines. However, the region is witnessing a significant increase in cancer incidence and mortality, with many patients being diagnosed at later stages when treatment options are limited, and survival rates are unfavorable [1,2]. Notably, the GCC region faces additional challenges in cancer care, as the average age of cancer diagnosis is younger compared to Western countries. In Oman, a recent report revealed that a substantial portion of colorectal cancer patients are below the age of 50 [3], and unfortunately, young cancer patients are often diagnosed at later stages with more aggressive forms of the disease [4,5,6]. This necessitates aggressive treatment and leads to complex side effects.

Generally, cancer patients diagnosed at a later stage experience various symptoms that significantly impact their quality of life, with pain being one of the most prominent. Unfortunately, access to adequate cancer pain relief remains a substantial problem worldwide. The 2015 Quality of Death Index ranked many Middle Eastern and East African countries at the bottom of the scale in terms of palliative care, including pain management [7]. Several factors were blamed for inadequate pain control, such as inadequate knowledge [8], lack of pain assessment due to nurses’ overload [9], reluctance to prescribe opioids by healthcare providers [10], and fear of opioid side effects and addiction [11,12]. Therefore, proper training of healthcare providers in cancer pain management is needed to improve their knowledge and skills and to consider the patient’s special needs at different ages and disease stages.

Nurses, being the largest group within the healthcare team, play a crucial role in cancer care. They often serve as the primary point of contact for patients and families. Nurses are instrumental in evaluating and managing cancer pain, and their level of knowledge, expertise, and attitudes significantly impact the quality of pain control [13]. However, research indicates that nurses working with cancer patients often lack the necessary training and knowledge for effective pain management. This leads to inadequate pain relief, increased patient suffering, and prolonged hospital stays [14,15]. Kelman’s theory of Knowledge Attitude/Brief Practice (KABP) emphasizes that both knowledge and attitude influence behavior, with knowledge serving as the foundation and behavior being driven by attitude [16]. Therefore, it is important to evaluate nurses’ knowledge and attitudes regarding cancer pain management in order to better understand their behavior.

This study aims to assess the general knowledge and attitude of nurses involved in the care of cancer patients in Oman regarding cancer pain assessment, management, and pharmacology. The findings of this study can inform policymakers and guide efforts to incorporate cancer pain management (CPM) education into university curricula and provide pain management workshops for nurses. Ultimately, this will help bridge the knowledge gaps and enhance the quality of care provided to cancer patients.

## 2. Materials and Methods

### 2.1. Study Design and Setting

A cross-sectional survey-based study was conducted at the national oncology center of the Royal Hospital, a tertiary hospital that serves the population in Oman. This study was approved by the ethical committee from the Ministry of Health, Oman.

### 2.2. Participants

In this cancer center, cancer nurses take care of all adult cancer patients regardless of their age. Therefore, all nurses who are currently providing care to adult cancer patients at the national cancer center were invited to participate through institutional email. The survey was distributed to 150 registered cancer nurses, and 118 of them responded to the survey (78% response rate). All participants consented before participating in this study.

### 2.3. Study Tool and Data Collection

The “knowledge and attitude survey regarding pain” (KASRP) was used to evaluate nurses’ knowledge in general without specifying the AYA patient population or their needs, as this is a baseline study that will be followed by more customized questionnaires for the AYA group. The questionnaire constitutes of two parts:

Part I: Sociodemographic characteristics (6 items), which include gender, age, education levels, years of clinical experience, years of experience caring for patients with cancer, and previous training sessions/workshops in pain management.

Part II: The “knowledge and attitude survey regarding pain” (KASRP).

The KASRP was created in 1987 by Ferrell and McCaffery and has been revised several times, with the most recent revision completed in 2014 [14]. It is derived from current guidelines and standards for pain management published by the American Pain Society, the AHCPR, and the World Health Organization (WHO). It is a validated questionnaire with a good reliability score of alpha r > 0.70 [14]. The KASRP questionnaire is freely available and is widely used in various languages and contexts throughout the world. It includes a 36-item questionnaire covering pain evaluation, pharmacologic and non-pharmacologic therapies, and pain management attitudes. There are 22 true or false questions and 15 multiple-choice questions. For each item on the NKASRP, knowledge scores were calculated by assigning a “1” for correct answers and a “0” for incorrect or blank responses, summing the scores, and calculating the mean scores for overall pain knowledge. The percentages of accurate answers for each item and the total points earned are also calculated by dividing the total number of correct responses by the total number of KASRP items (X/36). Each participant’s total score ranges from 0 (lowest) to 36 (the highest). Therefore, a higher score reflects a greater number of correct survey responses.

The levels of knowledge and attitude were classified as poor (<50), fair (50–70%), and good (≥70%). The ANOVA test was used to determine whether there are any significant associations between levels of knowledge and attitudes toward CPM and nurses’ demographic characteristics. (*p* < 0.05) was considered significant.

## 3. Results

### 3.1. Sample Characteristics

Table 1 summarizes the demographic characteristics of the 118 participants. The mean age was 39 years, with the majority of the participants being female (93%). Almost half of the participants (53%) had a diploma degree in nursing. The majority of nurses (77) (65%) had ≥11 years of experience. Regarding experience in caring for cancer patients, the majority (71%) had cared for cancer patients for less than five years. The majority of participants (82%) reported receiving pain management training, either during their nursing education, while working, or both.

### 3.2. Nurses’ Knowledge and Attitudes Regarding Pain Management

Table 2 presents the percentages of questionnaire items that were correctly answered by all the participating nurses. The mean rate of correct responses was 49.6%, which indicates poor knowledge of pain management among the nurses.

**Figure 1:**
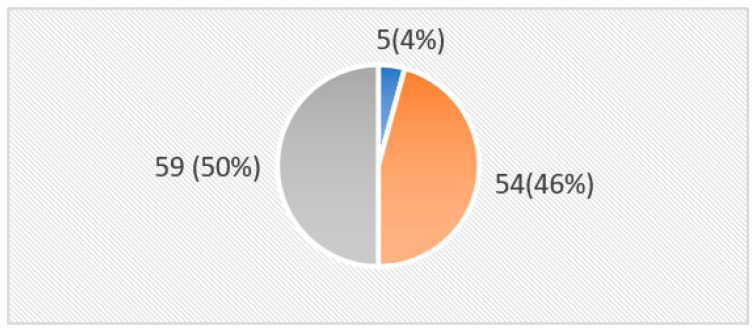
Total knowledge and attitude scores of nurses regarding cancer pain management. Grey: poor knowledge; Orange: fair knowledge; Blue: good knowledge.

On average, the nurses answered 17.86 out of 36 questionnaire items correctly (range = 9–33; SD = 3.970). Figure 1 classifies the levels of knowledge and attitude as Poor (<50%), Fair (50–70%), and Good (≥70%). The results show that half of the participants (50%) had a poor level of knowledge and attitude, while 46% had fair knowledge and attitude, and only five participants (4%) had a good level of knowledge and attitude.

The items with the highest percentage of accurate answers were: item number 7, “Combining analgesics that work by different mechanisms (e.g., combining an opioid with an NSAID) may result in better pain control with fewer side effects than using a single analgesic agent” (94.1%); item number 22 “Narcotic/opioid addiction is defined as a chronic neurobiological disease, characterized by behaviors that include one or more of the following: impaired control over drug use compulsive use, continued use despite harm, and craving” (90.7%); item number 16, “After an initial dose of opioid analgesic is given, subsequent doses should be adjusted in accordance with the individual patient’s response” (89.8%); and item number 24, “The recommended route administration of opioid analgesics for patients with brief, severe pain of sudden onset such as trauma or postoperative pain is” (80.5%). These questions were related to analgesics with various mechanisms of action, the concept of opioid addiction, and dose adjustment following the individual patient’s response, respectively. Only six out of the thirty-six questions had a greater than 70% correct answer rate, while the majority of the questions had a low percentage of accurate responses. For instance, 20 items received less than 50% correct answer rate (items number 1, 4, 5, 8, 10, 11, 13, 14, 17–20, 23, 26, 28, 30, 32, 33, 35, and 36). The questions related to pain assessment and pharmaceutical therapies had the lowest percentage of correct answers.

Table 3 shows no significant association between the nurses’ pain management knowledge and attitudes and any of their demographic characteristics, such as age, gender, education level, and nursing experience years (*p* > 0.05). However, the ANOVA test revealed that the education level, years of experience caring for people with cancer, and participation in previous cancer pain management training significantly impacted the knowledge and attitude levels (*p* < 0.05). Nurses who have a Master’s degree reported a higher knowledge level (67.78%) than those with diplomas or bachelor’s degrees. Nurses who had attended previous cancer pain management training during their nursing education and employment had a mean KASRP score of 54.27%, which was 10.46% higher than that of nurses who had not received any prior training. Furthermore, nurses with more than five years of experience in managing cancer pain had higher scores (53.55%) than those with less than five years of experience.

## 4. Discussion

Inadequate management of cancer pain and the inappropriate use of opioids in cancer patients are significant public health concerns [17]. Insufficient pain control can lead to preventable complications and escalate the cost of cancer care, while the misuse of opioids can result in severe side effects and addiction. Previous research has highlighted the critical role of healthcare professionals, particularly nurses, in ensuring the effective management of cancer pain [13]. This cross-sectional study represents the first attempt to assess the attitudes and knowledge of nurses regarding cancer pain management in Oman. This study offers valuable insights into the level of knowledge and attitudes among nurses working at the Royal Hospital’s national cancer care center. The findings are particularly relevant as the hospital is currently contemplating the establishment of palliative care services in response to the growing number of late-stage cancer patients. The findings reveal that the participating nurses have inadequate to moderate knowledge and attitudes regarding pain management.

This study revealed that the average KASPR knowledge score of the participants was 17.86 ± 3.97 (49.6% ± 11.0%), and only 4% of the nurses achieved a good knowledge and attitude score of ≥70%. These findings are consistent with other studies conducted in the Middle East, where knowledge scores ranged from 45.1% to 66% [18,19,20,21,22,23]. Even in the USA, the score was found to be 64.6% [24]

A recent systematic review identified the lowest mean knowledge and attitude scores reported in KASPR as 28.5% from Iran [23], while the highest was 75% from Norway [25]. The variations in scores can be attributed to factors such as differences in sampling methods, sample size, data collection instruments, study settings, and participant categorization.

Furthermore, this study indicated that the nurses’ total score was not influenced by factors such as age, gender, or years of nursing experience. However, it was significantly associated with their level of education, years of experience in caring for cancer patients, and previous participation in training sessions or workshops related to pain management. Previous research has consistently shown that higher scores are linked to longer work experience [26,27,28], higher qualifications [29,30], and prior training in cancer pain management [31].

This study’s findings highlighted the presence of disparities in nurses’ knowledge, beliefs, and pain management practices regarding pain management. Although a majority of the nurses acknowledged the subjective nature of pain and the importance of relying on the patient’s self-assessment, a considerable portion of participants expressed a belief in using a placebo to assess the authenticity of pain despite it being considered an unethical practice. Additionally, over two-thirds of the nurses relied on vital signs to determine the severity of pain and mistakenly assumed that stable vital signs indicated the absence of pain. A similar attitude was previously reported in the region [21,22].

In this study, only a small proportion of nurses (18.6%) demonstrated awareness of the potential risk of increasing morphine IV dosage by 5 mg/h, which could lead to a 1% increase in the incidence of respiratory depression in cancer patients. Furthermore, just 26.3% of nurses demonstrated familiarity with the signs of opioid physical dependency. Moreover, the majority of nurses (55.1%) agreed to withhold opioid analgesia and let the patient suffer, while an even larger majority (73.7%) agreed to prolong the patient’s suffering to identify the cause of pain, which aligns with findings from previous studies [32,33]. It is crucial for nurses to understand that prolonging a patient’s suffering to identify the cause of the pain is not beneficial to the patient, and using a placebo to assess pain is unethical. Additionally, nurses should also be aware of the potential risks associated with increasing opioid dosages and familiarize themselves with the signs of physical dependency.

This research underscores the limited understanding among nurses regarding drug pharmacology, dosage adjustment, addiction, and substance abuse. A majority of nurses (61%) hold the belief that patients with a history of substance abuse should be denied opioid analgesia, even when they are experiencing pain. This perspective is ethically unsound and clinically unjust, as all patients deserve access to appropriate pain management, irrespective of their past substance abuse.

Given this insufficiency in knowledge and attitude towards cancer pain management, it is essential to provide comprehensive education and training in this subject, tailored to the unique needs of cancer patients. This has become particularly important considering the plans to establish palliative care services in the country, aiming to address the needs of cancer patients in advanced stages, especially when considering the rising number of late-stage cancer diagnoses, particularly among young patients who tend to experience more complex pain, requiring higher doses of opioids compared to older patients with cancer [34,35]. Additionally, several studies have demonstrated that younger patients experience severe cancer pain at all stages of the disease, including active treatment, survivorship, and advanced or incurable stages, which are often underestimated and undertreated [36,37,38]. Collectively this highlights the growing demand for comprehensive and customized palliative care that addresses cancer patients’ diverse needs across different ages [6]. Initial attempts to integrate palliative care in Oman were inconsistent and limited in scope. While some palliative care topics were included in healthcare professional education curricula, a formal training program specifically focused on palliative care was lacking.

The low knowledge score might be due to a shortage of continuing medical education courses on such topics, which necessitates introducing such education [39]. Previous studies have demonstrated that cancer pain education leads to improved pain assessment, individualized treatment plans, effective symptom management, enhanced communication between healthcare providers and patients, shared decision-making, and improved adherence to pain medications [40,41,42,43]. Therefore, it is imperative to integrate cancer pain education into comprehensive cancer care. This step will help nurses deal with the complex nature of pain faced by cancer patients and improve their quality of life considering the multidimensional causes of cancer pain, such as emotional, spiritual, and physical. This is even crucial considering the increased incidence of cancer among young patients at advanced stages with their special needs and challenges. 

Numerous cancer centers worldwide have implemented educational initiatives in order to enhance nurses’ understanding of cancer pain management [40,41,42,43]. One prominent program is the End-of-Life Nursing Education Consortium (ELNEC) Core Curriculum, established in 2000 by the City of Hope Medical Center and the American Association of Colleges of Nursing (AACN). This curriculum has been introduced at national and international levels to educate nurses and healthcare professionals about end-of-life care, specifically addressing the special requirements of patients in that stage of life [44]. The curriculum includes a “train the trainer” program, enabling nurses to receive training and subsequently become trainers themselves in their respective countries. This could be the initial plan in Oman to improve cancer pain management and facilitates the implementation of regular training programs, such as continuous medical education, which should be mandatory for nurses and healthcare providers. Additionally, cancer care specialists advocate for the creation of specialized multidisciplinary teams for various age groups, particularly for young cancer patients, such as the AYA support team. These teams offer comprehensive care tailored to the specific needs of this population.

## 5. Limitations

The present study has several limitations that must be acknowledged. First, the research was conducted at only one hospital in Oman, making it difficult to generalize the findings to all nurses in the country. Second, the sampling strategy employed in this study may have led to selection bias, which could have impacted the results. Thirdly, the survey was distributed to nurses without any restriction on their access to the internet, allowing them to potentially search for or discuss the information before participating. Moreover, while this study primarily focused on nurses’ knowledge and attitudes toward pain treatment, their actual practices were not observed, and thus, more comprehensive monitoring of nursing practices is required. Moreover, this study did not track the frequency with which nurses cared for cancer patients, which could have impacted their pain-related knowledge. Lastly, information about the nurses’ postgraduate pain courses, including their content and duration, was not collected. Future research should address these limitations and assess their influence on pain-related knowledge.

## 6. Conclusions

In conclusion, the low level of knowledge and attitude among nurses in cancer pain management is a significant challenge in providing comprehensive cancer care. Adequate training of nurses in cancer pain management is essential to provide effective pain management and improve the quality of life of cancer patients. To build a comprehensive cancer care program for adolescents and young adults with cancer, a multidisciplinary team should be established to address their special needs and concerns. The team should consist of experts from different disciplines, including physicians, specialized nurses, social workers, and psychologists, to provide the necessary support and care. By improving the knowledge and skills of nurses and establishing a multidisciplinary team, we can ensure that cancer patients receive the best possible care and support during their treatment and recovery.

## Figures and Tables

**Table 1:** Demographic characteristics of participants (*n*= 118).

Characteristics	*n* (%)
**Gender**	
Female	110 (93)
Male	8 (7)
**Age**	
20–25	3 (3)
26–30	20 (18)
31–35	31 (26)
36–40	40 (34)
>40	24 (20)
**Education Level**	
Diploma	63 (53)
Bachelor	50 (42)
Master	5 (4)
**Clinical Experience (Years)**	
1–5	16 (14)
6–10	25 (21)
11–15	37 (31)
16–20	25 (21)
>20	15 (13)
**Years of Experience In Managing Cancer Pain**	
No experience (less than 1 year)	47 (40)
1–5 years	24 (20)
More than 5 years	47 (40)
**Previous Cancer Pain Management Training**	
None	22 (19)
During nursing education	13 (11)
While employed	42 (36)
Both during education and employment	41 (35)

**Table 2:** Correctly answered questions in the questionnaire.

Item Number	Item Content	The Correct Answer	Number of Correct Answers	%
True and False				
1	Vital signs are always reliable indicators of the intensity of a patient’s pain.	F	15	12.7
2	Because their nervous system is underdeveloped, children under two years of age have decreased pain sensitivity and limited memory of painful experiences.	F	67	56.8
3	Patients who can be distracted from pain usually do not have severe pain.	F	65	55.1
4	Patients may sleep in spite of severe pain.	T	19	16.1
5	Aspirin and other nonsteroidal anti-inflammatory agents are NOT effective analgesics for painful bone metastases.	F	30	25.4
6	Respiratory depression rarely occurs in patients who have been receiving stable doses of opioids over a period of months.	T	78	66.1
7	Combining analgesics that work by different mechanisms (e.g., combining an opioid with an NSAID) may result in better pain control with fewer side effects than using a single analgesic agent.	T	111	94.1
8	The usual duration of analgesia of 1–2 mg morphine IV is 4–5 h.	F	38	32.2
9	Research shows that promethazine (Phenergan) and hydroxyzine (Vistaril) are reliable opioid analgesic potentiators.	F	61	51.7
10	Opioids should not be used in patients with a history of substance abuse.	F	46	39
11	Morphine has a dose ceiling (i.e., a dose above which no greater pain relief can be obtained).	F	44	37.3
12	Elderly patients cannot tolerate opioids for pain relief.	F	79	66.9
13	Patients should be encouraged to endure as much pain as possible before using an opioid.	F	53	44.9
14	Children less than 11 years old cannot reliably report pain, so nurses should rely solely on the parent’s assessment of the child’s pain intensity.	F	54	45.8
15	Patients’ spiritual beliefs may lead them to think pain and suffering are necessary.	T	73	61.9
16	After an initial dose of opioid analgesic is given, subsequent doses should be adjusted in accordance with the individual patient’s response.	T	106	89.8
17	Giving patients sterile water by injection (placebo) is a useful test to determine if the pain is real.	F	52	44.1
18	Tramadol 50 mg PO is approximately equal to 5 mg of morphine PO.	T	35	29.7
19	If the source of the patient’s pain is unknown, opioids should not be used during the pain evaluation period, as this could mask the ability to correctly diagnose the cause of pain.	F	31	26.3
20	Anticonvulsant drugs such as gabapentin (Neurontin) produce optimal pain relief after a single dose.	F	53	44.9
21	Benzodiazepines are not effective pain relievers unless the pain is due to muscle spasms.	T	72	61
22	Narcotic/opioid addiction is defined as a chronic, neurobiological disease, characterized by behaviors that include one or more of the following: impaired control over drug use, compulsive use, continued use despite harm, and craving.	T	107	90.7
Multiple choice questions				
23	The recommended route of administration of opioid analgesics for patients with persistent cancer-related pain is:	Oral	30	25.4
24	The recommended route administration of opioid analgesics for patients with brief, severe pain of sudden onset, such as trauma or postoperative pain, is:	Intravenous	95	80.5
25	Which of the following analgesic medications is considered the drug of choice for the treatment of prolonged moderate to severe pain for cancer patients?	Morphine	84	71.2
26	Which of the following IV doses of morphine administered over a 4 h period would be equivalent to 30 mg of oral morphine given q 4 h?	Morphine 10 mg IV	39	33.1
27	Analgesics for postoperative pain should initially be given:	Around the clock on a fixed schedule	93	78.8
28	A patient with persistent cancer pain has been receiving daily opioid analgesics for 2 months. Yesterday, the patient was receiving morphine 200 mg/h intravenously. Today he has been receiving 250 mg/h intravenously. The likelihood of the patient developing clinically significant respiratory depression in the absence of new comorbidity is:	Less than 1%	22	18.6
29	The most likely reason a patient with pain would request increased doses of pain medication is:	The patient is experiencing increased pain	80	67.8
30	Which of the following is useful for the treatment of cancer pain?	All the above	54	45.8
31	The most accurate judge of the intensity of the patient’s pain is:	The patient	70	59.3
32	Which of the following describes the best approach for cultural considerations in caring for patients in pain?	The patient should be individually assessed to determine cultural influence	53	44.9
33	How likely is it that patients who develop pain already have an alcohol and/or drug abuse problem?	5–15%	39	33.1
34	The time to peak effect for morphine given IV is:	15 min	82	69.5
35	The time to peak effect for morphine given orally is:	1–2 h	47	39.8
36	Following the abrupt discontinuation of an opioid, physical dependence is manifested by the following:	Sweating, yawning, diarrhea, and agitation with patients when the opioid is abruptly discontinued	31	26.3

**Table 3:** Nurses’ mean scores based on their characteristics.

Characteristic	Score (Mean)	SD	Std. Error	*p*-Value
**Gender**				
Female	49.39	11.107	1.059	0.404
Male	52.78	9.960	3.521	
**Age**				
20–25	43.52	4.243	2.450	0.147
26–30	44.44	7.959	1.780	
31–35	51.52	11.314	2.032	
36–40	50.49	12.966	2.050	
>40	50.81	8.764	1.789	
**Education Level**				
Diploma	47.35	8.473	1.067	0.000
Bachelor	50.67	11.952	1.690	
Master	67.78	13.693	6.124	
**Clinical Experience (Years)**				
1–5	43.06	7.244	1.811	0.077
6–10	51.22	12.605	2.521	
11–15	49.62	12.528	2.060	
16–20	52.78	9.351	1.870	
>20	48.70	7.847	2.026	
**Years of Experience In Managing Cancer Pain**				
No experience	46.63	9.604	1.401	0.006
Less than 5 years	47.80	9.829	2.006	
More than 5 years	53.55	11.922	1.739	
**Previous Cancer Pain Management Training**				
None	43.81	11.240	2.396	0.002
During nursing education	47.65	8.990	2.493	
While employed	48.74	11.283	1.741	
Both during education and employment	54.27	9.584	1.497	

## Data Availability

The author can provide the data upon request.
